# Thinking about thinking: A coordinate-based meta-analysis of neuroimaging studies of metacognitive judgements

**DOI:** 10.1177/2398212818810591

**Published:** 2018-11-13

**Authors:** Anthony G. Vaccaro, Stephen M. Fleming

**Affiliations:** 1 Division of Psychology and Language Sciences, University College London, London, UK; 2 Yale Child Study Center, Yale School of Medicine, New Haven, CT, USA; 3 Wellcome Centre for Human Neuroimaging, University College London, London, UK; 4 Max Planck UCL Centre for Computational Psychiatry and Ageing Research, University College London, London, UK

**Keywords:** Confidence, decision-making, mentalising, meta-analysis, metacognition, metamemory, prefrontal cortex

## Abstract

Metacognition supports reflection upon and control of other cognitive processes. Despite metacognition occupying a central role in human psychology, its neural substrates remain underdetermined, partly due to study-specific differences in task domain and type of metacognitive judgement under study. It is also unclear how metacognition relates to other apparently similar abilities that depend on recursive thought such as theory of mind or mentalising. Now that neuroimaging studies of metacognition are more prevalent, we have an opportunity to characterise consistencies in neural substrates identified across different analysis types and domains. Here we used quantitative activation likelihood estimation methods to synthesise findings from 47 neuroimaging studies on metacognition, divided into categories based on the target of metacognitive evaluation (memory and decision-making), analysis type (judgement-related activation, confidence-related activation, and predictors of metacognitive sensitivity), and, for metamemory judgements, temporal focus (prospective and retrospective). A domain-general network, including medial and lateral prefrontal cortex, precuneus, and insula was associated with the level of confidence in self-performance in both decision-making and memory tasks. We found preferential engagement of right anterior dorsolateral prefrontal cortex in metadecision experiments and bilateral parahippocampal cortex in metamemory experiments. Results on metacognitive sensitivity were inconclusive, likely due to fewer studies reporting this contrast. Finally, by comparing our results to meta-analyses of mentalising, we obtain evidence for common engagement of the ventromedial and anterior dorsomedial prefrontal cortex in both metacognition and mentalising, suggesting that these regions may support second-order representations for thinking about the thoughts of oneself and others.

## Introduction

Metacognition allows reflection upon and control of other cognitive processes such as perception, decision-making, and memory (Metcalfe and Shimamura, 1996). Efforts to quantify metacognition have focussed on how people judge their performance (second-order judgements) in a variety of domains (Fleming and Lau, 2014). For instance, in perceptual decision-making tasks, a first-order discrimination is made about a stimulus (e.g. orientation of a grating), followed by a second-order assessment of confidence of whether the first-order discrimination is likely to be correct. Effective metacognitive monitoring is important for behavioural control, such as when one recognises a poor decision and pursues an alternative course of action. Accounting for deficits in metacognitive function may shed light on the causes of a lack of insight into neuropsychiatric disorders and reveal possible diagnostic and therapeutic options which target metacognitive abnormalities (David et al., 2014; Koren et al., 2006).

Despite a central role for metacognition in the monitoring and control of behaviour, the relevant neurocognitive architecture supporting metacognition remains poorly understood. Initial neuropsychological studies pointed to the importance of the frontal lobe in second-order judgements about memory performance (Janowsky et al., 1989; Shimamura and Squire, 1986), with selective deficits in metacognition observed in conditions such as Korsakoff’s syndrome associated with frontal atrophy. In parallel, studies of performance monitoring identified neural signals involved in error monitoring originating in posterior medial frontal cortex (Dehaene et al., 1994; Gehring et al., 1993). Since the introduction of these seminal studies, a standard approach leverages modern neuroimaging methods to identify neural correlates of metacognitive judgements across different tasks, primarily recognition memory (metamemory), and perceptual and value-based decision-making (which we collectively refer to here as ‘metadecision’). Such research has confirmed the involvement of a frontoparietal network in metacognition (Fleming and Dolan, 2014) and begun to assign distinct computational roles to elements within this network (Bang and Fleming, 2018; Kepecs et al., 2008; Kiani and Shadlen, 2009; Miyamoto et al., 2018).

A complementary but hitherto distinct perspective on the brain basis for metacognition is provided by studies of theory of mind (ToM) – the capacity to understand others’ mental states and to appreciate that these may differ from our own. Carruthers’ interpretive sensory-access (ISA) theory proposes that self-directed metacognition relies on turning a specialised circuit for mindreading on ourselves, to indirectly infer our state of mind (Carruthers, 2009, 2011; Frith, 2012). This view is related to a recent proposal that confidence in our own actions is formed via a second-order evaluation of a coupled but distinct decision system, computationally equivalent to inferring the performance of another actor (Fleming and Daw, 2017). Indirect evidence for this view has been found in developmental studies that reveal the ability to explicitly monitor self-performance (using confidence ratings) is gained at around the same age (4–5 years old) as children begin to pass false-belief tests (Hembacher and Ghetti, 2014; Lockl and Schneider, 2007). Neuroimaging studies of mentalising have also highlighted a frontoparietal network, with meta-analyses identifying anterior dorsal medial prefrontal cortex (mPFC), bilateral temporoparietal junction (TPJ), and precuneus as key nodes (Amodio and Frith, 2006; Frith and Frith, 1999; Molenberghs et al., 2016; Schurz et al., 2014). However, given that relatively less is known about the neural basis of metacognition than ToM, whether metacognition and ToM (and mentalising more specifically) share neural substrates remains an open question (Lombardo et al., 2010; Schilbach et al., 2012; Valk et al., 2016).

When drawing inferences about the architecture of metacognition from individual studies, it is important to consider the class of second-order judgement being elicited (Schwartz and Diaz, 2014). Metacognitive judgements can be subdivided by both domain and temporal focus – retrospective or prospective (Fleming and Dolan, 2014). For instance, judgements of confidence or monitoring for errors are *retrospective* judgements of performance, whereas *prospective* judgements (typically used in metamemory tasks) include ‘feelings of knowing’ (FOKs) and ‘judgements of learning’ (JOLs) that refer to one’s future task performance. Lateral and medial aspects of prefrontal cortex (PFC) have been suggested to support retrospective and prospective judgements, respectively (Fleming and Dolan, 2014; Pannu et al., 2005). However, direct neuroimaging evidence for a distinction between different judgement types is surprisingly limited. In one of the few studies to directly compare activation related to retrospective confidence ratings and prospective FOKs, Chua et al. (2009) found that prospective judgements are associated with medial parietal and medial temporal lobe (MTL) activation, whereas retrospective judgements were related to inferior prefrontal activity. However, the same study also found that both forms of metamemory activated common regions of medial and lateral PFC, and mid-posterior areas of cingulate cortex, indicating differences may be of degree rather than of kind.

An interrelated question is whether metacognition relies on a common, domain-general resource that is recruited to evaluate performance across a variety of first-order tasks, or whether metacognition is supported by domain-specific components. Current behavioural evidence for a domain-general resource is mixed: some studies find that efficient metacognition in one task predicts good metacognition in another (Ais et al., 2016; Faivre et al., 2018; McCurdy et al., 2013; Schraw, 1996; Song et al., 2011), whereas others argue for a separation between metacognitive abilities (Baird et al., 2013; Fitzgerald et al., 2017; Garfinkel et al., 2016; Morales et al., 2018; Kelemen et al., 2000). Moreover, a correlation in behavioural measures does not necessarily mean they share the same (neural) resource, as even correlated metacognitive functions can be associated with distinct neurostructural profiles (Mccurdy et al., 2013; Rouault et al., in press). Recent neuroimaging studies have highlighted both domain-general and domain-specific neural substrates (Baird et al., 2013, 2015; Chiou et al., 2011; Fleming et al., 2014; Morales et al., 2018; Valk et al., 2016), with metamemory broadly hypothesised to recruit parietal and midline prefrontal regions, while metadecision recruits frontal regions including anterior cingulate cortex (ACC), insula, and lateral anterior prefrontal cortex (aPFC; Baird et al., 2013). However, direct comparisons between domains remain rare.

In sum, the neurocognitive architecture of metacognition remains underdetermined, partly due to study-specific differences in task domain and type of metacognitive judgement under study. Now that neuroimaging studies of metacognition are more prevalent, we have an opportunity to characterise consistencies in neural substrates of metacognition identified across different studies and task domains. In this study, we used activation likelihood estimation (ALE; Eickhoff et al., 2009) to perform meta-analyses of the current neuroimaging literature on metacognition across the two most studied domains: decision-making and memory. We also sought to analyse distinctions between different aspects of metacognitive judgements (e.g. their level and sensitivity to performance) and, within metamemory studies, their temporal focus (prospective vs retrospective). Our study thus builds on and extends a previous meta-analysis that focussed on retrospective confidence judgements about memory (White et al., 2014). Finally, we also compare the results of our meta-analysis of self-directed metacognition to networks engaged during mentalising about others.

## Methods

### Identifying candidate studies

Candidate studies for inclusion were initially identified from a PubMed search using the following string: (metacognition OR metamemory OR metacognitive OR ‘decision confidence’ OR ‘memory confidence’ OR ‘feeling of knowing’ OR ‘judgment of learning’ OR ‘error awareness’ OR ‘tip of the tongue’) AND (MRI OR fMRI OR ‘magnetic resonance imaging’). This string returned 169 records on 25 March 2018. The following selection criteria were used to identify studies for further evaluation: (1) studies reported in peer-reviewed journals published in English; (2) use of functional or structural MRI with associated behavioural measurements; (3) the task involved a metacognitive judgement by the subject; (4) stereotactic three-dimensional (3D) coordinates were reported from whole-brain analyses; (5) the study reported a contrast that fell into at least one of our analysis categories of interest (judgement-related activation, confidence-related activation or neural correlates of metacognitive sensitivity; refer section ‘Analysis’); and (6) the study includes data from healthy participants. Our meta-analysis differed from that of White et al. (2014) in which we required an explicit metacognitive judgement from the subject (their ‘Type B’ studies), while excluding studies which solely manipulated environmental uncertainty (their ‘Type A’ category).

From studies that met these criteria, we further limited our cohort to the two most prevalent domains in the literature: metacognition of decision-making (metadecision) and metacognition of memory (metamemory). Other less frequently studied tasks (i.e. metacognition of emotion) were excluded from analysis. Of these 169 initial results, 34 met our criteria. To ensure our search was comprehensive, we also consulted studies cited in review chapters from ‘The Cognitive Neuroscience of Metacognition’ book (Fleming and Frith, 2014) and searched the following string on Google Scholar: (metacognition OR metacognitive OR ‘error awareness’ OR ‘feeling of knowing’ OR ‘memory confidence’ OR ‘decision confidence’ OR metamemory) AND (fMRI OR MRI). These two sources resulted in an additional 13 studies that met our criteria.

### Final corpus

The final corpus of 47 studies included a total of 88 analysis contrasts, 739 activation foci, and 2215 participants (see Table S1 in Supplementary Materials for full details). The number of participants in each study ranged from 11 to 191 with a mean of 47.13. One of the included studies (Hester et al., 2009) reported data collected from patient populations, but only the results from the control group were included. Coordinates reported in Talairach space were converted to Montreal Neurological Institute coordinates using the algorithm in the GingerALE software (Eickhoff et al., 2009).

### Analysis

Activation-level estimation analyses were run using GingerALE (version 2.3.6) software (Eickhoff et al., 2009, 2012). The most recent instantiation of the ALE algorithm tests for clustering of peak foci from different experiments against an ALE null distribution created by randomly redistributing the same number of foci throughout the brain volume. In a typical study, the same group of subjects will contribute data to multiple statistical contrasts, and consequently, the activation patterns produced by different contrasts do not constitute independent observations. We therefore organised reported foci according to subject group (rather than contrast) and used the modified ALE algorithm to address this issue, as recommended by Turkeltaub et al. (2012). All coordinate files used in the analysis are available for download at https://github.com/metacoglab/VaccaroFleming.

Included activation foci were smoothed using a Gaussian kernel whose size depended on the sample size (larger samples result in a smaller smoothing kernel; Eickhoff et al., 2009, 2012). Multiple-comparisons correction was applied at the cluster level at a family-wise error-corrected threshold of *p* < .05, 5000 permutations, and a cluster-forming threshold of *p* < .001 uncorrected. The resulting statistical maps indicate areas of the brain where convergence between activation foci is greater than would be expected by chance (i.e. a null distribution of clusters). We followed similar methods to those used in other recent ALE studies (Garrison et al., 2013; Pollack and Ashby, 2018; Sokolowski et al., 2017). 3D statistical maps can be viewed at https://neurovault.org/collections/4238/.

Activations were labelled using a combination of group atlases and anatomical landmarks. For greater specificity in labelling clusters obtained within PFC, we also referenced coordinates against the Oxford atlases included in the FMRIB Software Library (FSL; Jenkinson et al., 2012). These atlases are derived from studies using diffusion-tensor imaging to subdivide regions sharing common connectivity fingerprints, including dorsal and ventral PFC, cingulate cortex, and orbitofrontal cortex (Neubert et al., 2014, 2015; Sallet et al., 2013).

### Classification of contrasts of interest

In addition to classifying activation foci by domain (metadecision and metamemory), we also subdivided contrasts by analysis type, collapsing across domains. We identified three common contrasts of interest (Chua et al., 2014). ‘Judgment-related activity’ refers to contrasts comparing the requirement for a metacognitive judgement against a baseline or control condition. ‘Parametric effect of confidence’ refers to contrasts identifying activations that scale with the metacognitive judgement, such as a negative/positive parametric effect of JOL or confidence rating. ‘Metacognitive sensitivity’ refers to analyses identifying differences in the extent to which metacognitive judgements track objective task performance. Finally, for metamemory judgements, we also divided judgements by temporal focus (prospective and retrospective), while collapsing over contrast type. The one exception to this tripartite classification of analyses was for studies using the ‘Error Awareness Task’ (Hester et al., 2005). In this task, subjects are asked to detect each time they make an error on a go/no-go task, which is typically only achieved around 70% of the time. This feature of the task permits a contrast between ‘aware’ (reported) and ‘unaware’ (unreported) errors. We classified this contrast as both judgement and confidence related, as it reflects the deployment of a metacognitive judgement and lowered confidence in performance.

We conducted eight distinct meta-analyses: (1) all metacognition-related activations, collapsing over both domain and analysis type, (2) judgement-related activations, collapsing over domain, (3) parametric effects of confidence, collapsing over domain, (4) correlates of metacognitive sensitivity, collapsing over domain, (5) metamemory-related activations, collapsing over contrast type, (6) metadecision-related activations, collapsing over contrast type, (7) prospective metamemory-related activations, collapsing over contrast type, and (8) retrospective metamemory-related activations, collapsing over contrast type.

### Comparing metacognition and ToM

To compare metacognition-related activations in our de novo meta-analysis to those associated with ToM, we obtained the ‘reverse inference’ map associated with the term ‘mentalising’ from Neurosynth (www.neurosynth.org, accessed June 2018). Neurosynth uses text-mining combined with meta-analysis to generate a large database of mappings between neural and cognitive states (Yarkoni et al., 2011). A reverse inference map displays brain regions that are preferentially related to mentalising over and above other terms in the database (i.e. that show a high posterior probability P(mentalising|activation)). The map is corrected for multiple comparisons using a false discovery rate (FDR) approach at *p* < 0.01. We computed the overlap between the Neurosynth mentalising map and our composite map of metacognition-related activity to examine common and distinct regional engagement. Note that while both maps are corrected for multiple comparisons across the whole-brain volume, the numerical values and thresholds are not comparable, as they are obtained via different meta-analytic methods (ALE for metacognition and multilevel kernel density analysis (MKDA) for mentalising).

## Results

### Composite meta-analysis of metacognition-related activity

**Figure 1.**
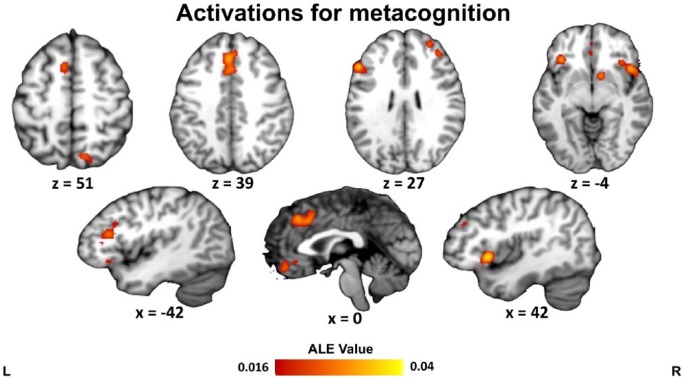
ALE results for all studies on metacognition. Clusters are displayed in MNI standard space. Multiple-comparisons correction was applied at the cluster level at a family-wise error-corrected threshold of *p* < .05, 5000 permutations, and a cluster-defining threshold of *p* < .001 uncorrected.

Collapsing across all 47 studies (739 foci), eight significant clusters were identified: in posterior mPFC (paracingulate gyrus/dorsal ACC), left and right insula/inferior frontal gyrus, left and right dorsolateral PFC, ventromedial PFC, right ventral striatum, and right dorsal precuneus (Table 1 and Figure 1). Notably, the activation in right dorsolateral PFC was more anterior (anterior border y = 56) than that on the left (anterior border y = 40).

**Table 1.** ALE meta-analysis of all metacognition-related activations (FWE cluster-level correction *p* < .05; cluster-defining threshold *p* < .001 uncorrected, and 5000 permutations).

Cluster	Peak coordinate (MNI)			Volume (mm^3^)	Region	Maximum ALE value
	x	y	z			
1	−2	30	38	5096	L/R posterior medial frontal cortex	0.0331
2	44	16	0	4424	R insula/inferior frontal gyrus	0.0398
3	−50	24	28	3656	L dorsolateral prefrontal cortex	0.0349
4	−36	28	−6	1432	L insula/inferior frontal gyrus	0.0318
5	28	50	26	1160	R anterior dorsolateral prefrontal cortex	0.0245
6	−2	44	−12	1152	L/R ventromedial prefrontal cortex	0.0275
7	12	−66	54	1112	R dorsal precuneus	0.0275
8	10	8	−2	952	R ventral striatum	0.0290

L: left; R: right; ALE: activation likelihood estimation; MNI: Montreal Neurological Institute.

### Meta-analysis of judgement-related activity

We next separated activation foci by contrast type. A common distinction in the metacognition literature is between activations related to the requirement for a metacognitive judgement (judgement-related activity vs baseline/control) and those tracking judgement level (e.g. parametric effect of confidence). The analysis of judgement-related activity included 12 studies (94 foci). This analysis did not yield consistent clusters, perhaps reflecting a lack of power given that between 17–20 experiments are typically considered necessary for a well-powered neuroimaging meta-analysis (Muller et al., 2018). For completeness, in Supplementary Materials, we include an exploratory analysis of judgement-related effects at *p* < .001, uncorrected, minimum cluster size 200 mm^3^ (Table S2 and Figure S1).

### Meta-analysis of parametric effects of confidence level

**Figure 2.**
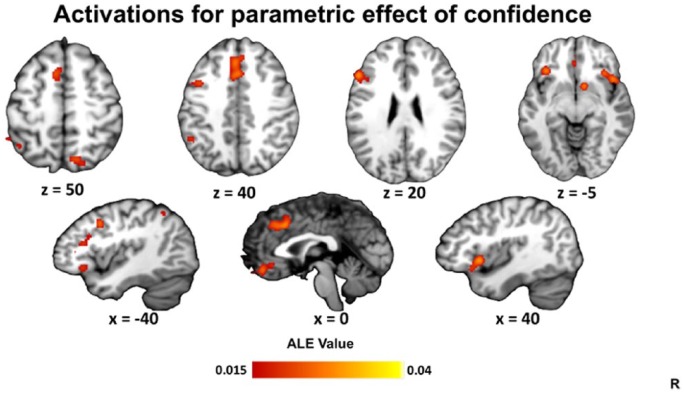
ALE results for parametric effects of confidence. Clusters are displayed in MNI standard space. Multiple-comparisons correction was applied at the cluster level at a family-wise error-corrected threshold of *p* < .05, 5000 permutations, and a cluster-defining threshold of *p* < .001 uncorrected.

We next examined parametric contrasts for activations co-varying positively or negatively with metacognitive ratings (e.g. the level of confidence or magnitude of JOL). This analysis included 36 studies (606 foci). Nine significant clusters were identified: in posterior medial frontal cortex, left and right insula/inferior frontal gyrus, left dorsolateral PFC, ventromedial PFC, right dorsal precuneus, left lateral parietal cortex, and right ventral striatum (Table 2 and Figure 2).

**Table 2.** ALE meta-analysis of parametric confidence level–related activations (FWE cluster-level correction *p* < .05; cluster-defining threshold *p* < .001 uncorrected, and 5000 permutations).

Cluster	Peak coordinate (MNI)			Volume (mm^3^)	Region	Maximum ALE value
	x	y	z			
1	0	20	38	4784	L/R posterior medial frontal cortex	0.0281
2	44	16	0	4480	R insula/inferior frontal gyrus	0.0397
3	−50	24	28	3832	L dorsolateral prefrontal cortex	0.0348
4	−2	44	−12	1576	L/R ventromedial prefrontal cortex	0.0275
5	−34	26	−4	1432	L insula/inferior frontal gyrus	0.0272
6	12	−66	54	1408	R dorsal precuneus	0.0273
7	10	8	−2	1200	R ventral striatum	0.0290
8	−42	−54	48	1008	L lateral parietal cortex	0.0196
9	−40	10	38	872	L dorsolateral prefrontal cortex	0.0248

L: left; R: right; ALE: activation likelihood estimation.

### Meta-analysis of metacognitive sensitivity

Our final contrast type related to metacognitive sensitivity – the extent to which confidence effectively tracks task performance across trials. A high degree of metacognitive sensitivity is obtained when people ascribe high confidence to correct decisions, and low confidence to incorrect decisions. Because sensitivity is a property of multiple trials, it is typically analysed as a between-subjects variable.

A total of 11 studies in our corpus reported results pertaining to metacognitive sensitivity (61 foci). With cluster-correction, this analysis did not yield any consistent clusters, again consistent with a lack of power due to a limited number of studies. For completeness, in Supplementary Materials, we include an exploratory analysis of sensitivity-related effects at *p* < .001, uncorrected, minimum cluster size 200 mm^3^ (Table S2 and Figure S1).

### Composite meta-analysis of metadecision-related activity

**Figure 3.**
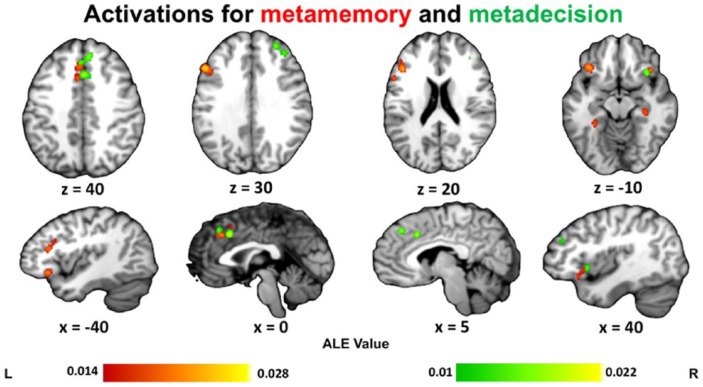
ALE results for all studies on metamemory (red) and metadecision (green). Clusters are displayed in MNI standard space. Multiple-comparisons correction was applied at the cluster level at a family-wise error-corrected threshold of *p* < .05, 5000 permutations, and a cluster-defining threshold of *p* < .001 uncorrected.

We next turned to the distinction between metacognition-related activations across domains (metadecision and metamemory) while collapsing over contrast type. For metadecision, we identified 20 studies (211 foci). Five clusters were found: one in right anterior dorsolateral PFC, two in posterior medial frontal cortex, and two in right insula/inferior frontal gyrus. In line with previous observations in the literature, we found that activations for metadecision were predominantly lateralised to the right hemisphere (Fleming and Dolan, 2014; Schmitz et al., 2004) (Table 3 and Figure 3).

**Table 3.** ALE meta-analysis of metadecision-related activations (FWE cluster-level correction *p* < .05; cluster-defining threshold *p* < .001 uncorrected, and 5000 permutations).

Cluster	Peak coordinate (MNI)			Volume (mm^3^)	Region	Maximum ALE value
	x	y	z			
1	26	48	28	1336	R anterior dorsolateral prefrontal cortex	0.0186
2	6	38	42	1232	L/R posterior medial frontal cortex	0.0172
3	32	20	−12	1056	R insula	0.0174
4	2	20	38	832	L/R posterior medial frontal cortex	0.0217
5	44	14	0	800	R insula/inferior frontal gyrus	0.0196

L: left; R: right; ALE: activation likelihood estimation.

### Composite meta-analysis of metamemory-related activity

Collapsing across contrast type, the ALE meta-analysis of metamemory included 30 studies (528 foci). Six significant clusters were identified: in left dorsolateral PFC, posterior mPFC (paracingulate gyrus), left and right insula/inferior frontal gyrus, and left and right parahippocampal gyrus (Table 4 and Figure 3).

**Table 4.** ALE meta-analysis of metamemory-related activations (FWE cluster-level correction *p* < .05; cluster-defining threshold *p* < .001 uncorrected, and 5000 permutations).

Cluster	Peak coordinate (MNI)			Volume (mm^3^)	Region	Maximum ALE value
	x	y	z			
1	−50	24	28	3656	L dorsolateral prefrontal cortex	0.0279
2	−2	28	36	2128	L/R posterior medial frontal cortex	0.0240
3	44	18	−2	2048	R insula/inferior frontal gyrus	0.0227
4	34	−28	−14	1376	R parahippocampal gyrus	0.0232
5	−36	26	−8	1224	L insula/inferior frontal gyrus	0.0245
6	−28	−38	−14	808	L parahippocampal gyrus	0.0217

L: left; R: right; ALE: activation likelihood estimation.

### Meta-analysis comparing prospective and retrospective metamemory judgements

**Figure 4.**
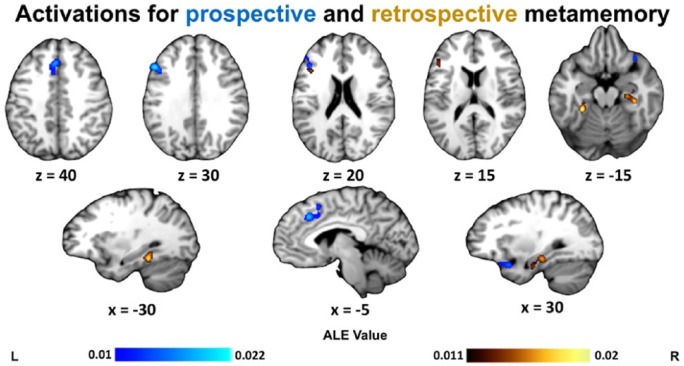
ALE results for contrasts of prospective metamemory (blue) and of retrospective metamemory (yellow). Clusters are displayed in MNI standard space. Multiple-comparisons correction was applied at the cluster level at a family-wise error-corrected threshold of *p* < .05, 5000 permutations, and a cluster-defining threshold of *p* < .001 uncorrected.

Within metamemory studies, we next examined possible differences in activation profile associated with prospective and retrospective metamemory judgements (Chua et al., 2009). Prospective judgements (such as JOLs or FOKs) included 14 studies with 232 foci, and retrospective judgements (such as recognition confidence) included 17 studies with 287 foci. The prospective analysis yielded three clusters: in posterior mPFC, left dorsolateral PFC, and right insula. The retrospective analysis revealed three clusters: in bilateral parahippocampal cortex and left inferior frontal gyrus (Table 5 and Figure 4).

**Table 5.** ALE meta-analysis of prospective and retrospective metamemory-related activations (FWE cluster-level correction *p* < .05; cluster-defining threshold *p* < .001 uncorrected, 5000 permutations).

Cluster	Peak coordinate (MNI)			Volume (mm^3^)	Region	Maximum ALE value
	x	y	z			
Prospective						
1	−2	28	36	2216	L/R posterior medial frontal cortex	0.0218
2	−50	24	28	2056	L dorsolateral prefrontal cortex	0.0224
3	30	14	−20	1040	R insula/inferior frontal gyrus	0.0152
Retrospective						
1	34	−28	−16	1632	R parahippocampal gyrus	0.0185
2	−28	−38	−14	1400	L parahippocampal gyrus	0.0203
3	−42	22	18	784	L inferior frontal gyrus	0.0156

L: left; R: right; ALE: activation likelihood estimation.

### Comparing metacognition and ToM

Finally, we examined a potential overlap between metacognition- and ToM-related activations by comparing our composite metacognition map (Figure 1) to a meta-analysis of ToM. ToM-related regions were obtained from the ‘reverse inference’ map for the term ‘mentalising’ in Neurosynth, which identifies regions that are preferentially associated with ToM over and above other terms in the database.

**Figure 5.**
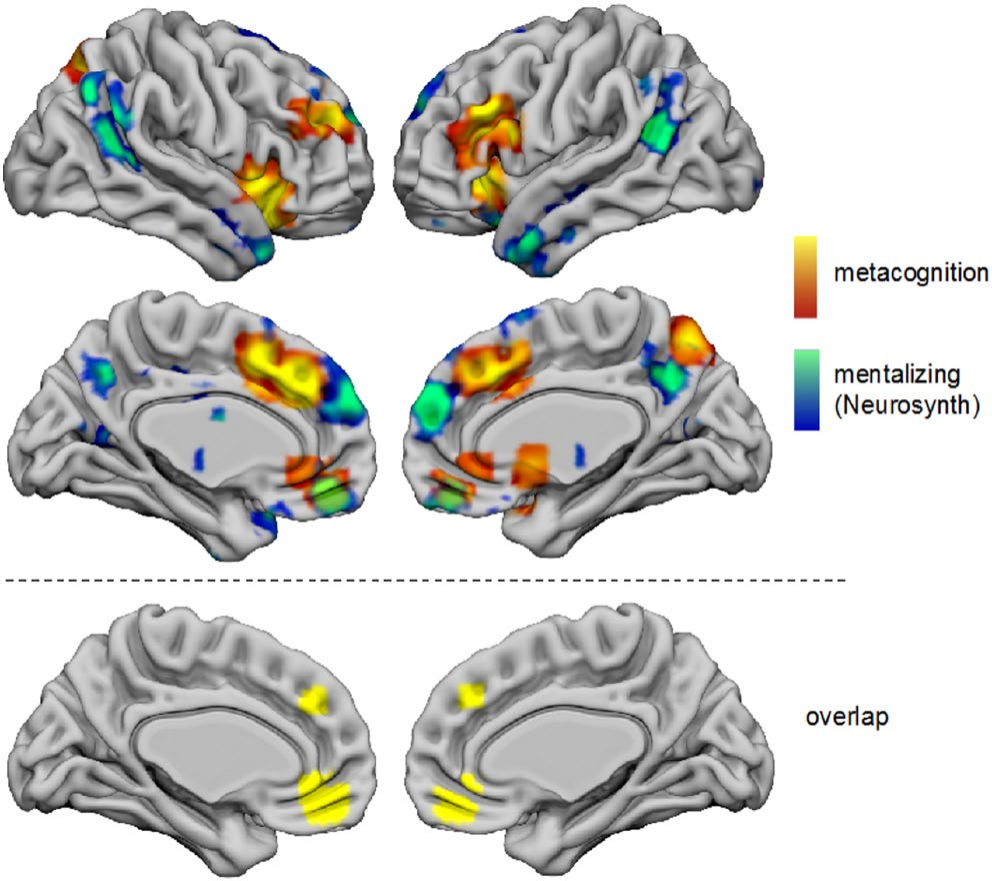
ALE results for all studies on metacognition (from Figure 1, hot colours) as compared to the Neurosynth reverse inference map for the term ‘mentalising’ (cool colours). Clusters are displayed in a 3D rendering of MNI standard space.

Figure 5 shows the two maps overlaid on the same cortical surface projection created using Surf Ice (https://www.nitrc.org/projects/surfice/). Activations for both metacognition and ToM were observed in mPFC and precuneus, with ToM activations tending to be anterior and ventral to metacognition-related activations. There was clear overlap between metacognition and ToM in vmPFC (cluster centre of mass (–3, 45, –12)) and a region of mid-dorsomedial PFC ((–4, 40, 34); Figure 5, bottom row). Unique activations for metacognition were observed in dorsolateral PFC, insula, and lateral parietal cortex; unique activations for ToM were observed in TPJ and temporal pole.

## Discussion

Deficits in metacognition – the ability to reflect on our cognitive processes – have clear and important consequences for functional capacity and quality of life, and are often found in psychiatric and neurological conditions (David et al., 2014; Koren et al., 2006). Metacognition has been considered a higher brain function that depends on the integrity of prefrontal and parietal association cortex (Shimamura, 2000) and that is particularly well-developed in humans compared to other animals (Metcalfe, 2008). However, the underlying neurocognitive architecture supporting metacognitive abilities remains poorly understood. By comparing the neural basis of metacognition across different judgement types (e.g. prospective versus retrospective judgements of performance) and tasks (e.g. decision-making and memory), we aimed to provide insight into the types of neurocognitive architecture (e.g. domain-general or domain-specific) that support human metacognition. In turn, we hope progress on this issue will aid in understanding the aetiology of metacognitive deficits.

Here, we present a first meta-analysis of the current neuroimaging literature on explicit metacognitive judgements of performance. We used quantitative ALE methods to synthesise findings from 47 structural or functional neuroimaging studies on metacognition, divided into categories based on domains (metamemory and metadecision), analysis type (judgement-related activity, parametric effect of confidence, and metacognitive sensitivity), and, for metamemory judgements, temporal focus (prospective and retrospective). We also compared our results on self-directed metacognition to those obtained in a previous meta-analysis of ToM, motivated by theoretical proposals that self-knowledge partly depends on co-opting machinery that originally evolved for mentalising about others (Carruthers, 2009, 2011; Fleming and Daw, 2017; Frith, 2012).

In a composite meta-analysis collapsing over both analysis type and domain, we found consistent involvement of a frontoparietal network. Previous reviews have highlighted the specific contribution of a network centred on medial/lateral aPFC in metacognition (Fleming and Dolan, 2014; Grimaldi et al., 2015; Metcalfe and Schwartz, 2015). We found evidence in line with this view, with metacognition-related activations in posterior mPFC, ventromedial PFC and bilateral aPFC/dorsolateral PFC. Notably, the lateral PFC activations were asymmetric: left lateral PFC was more posterior and corresponded closely to area 44d from the atlas of Neubert et al. (2014); the right lateral PFC cluster was more anterior, corresponding to Neubert et al.’s area 46. In addition to these prefrontal activations, we also observed the involvement of bilateral insula and dorsal precuneus. This is consistent with an emerging view that the parietal cortex, particularly precuneus, supports metacognition in concert with the PFC (Mccurdy et al., 2013; Simons et al., 2010). The insula, together with posterior mPFC has been implicated in error processing and error awareness (Bonini et al., 2014; Garavan et al., 2003; Ridderinkhof et al., 2004; Taylor et al., 2007; Ullsperger et al., 2010), and is a hub for interoception (Craig, 2009), thought to be a key modulator of, or input to, metacognitive appraisal (Allen et al., 2016; Stephan et al., 2016).

### Subcomponents of metacognition

We next turn to the review results for each contrast type separately. Previous studies have drawn a distinction between activations tracking the requirement for a metacognitive judgement relative to a baseline or control condition and those parametrically tracking the *level* of the judgement (e.g. high vs low confidence). For judgement-related activity, we did not find any consistent regions across the studies, likely due to this analysis being underpowered, given the relatively fewer studies reporting results for this contrast. In contrast, regions parametrically tracking confidence level were widespread and highlight a similar network to that found in the composite analysis (posterior mPFC, bilateral insula, right dorsal precuneus, ventral striatum, left posterior dorsolateral PFC, and ventromedial PFC), suggesting parametric effects were a predominant driver of the overall pattern. Parametric effects of confidence in ventromedial PFC are consistent with recent findings that perigenual ACC tracks determinants of subjective confidence arising from multiple sources during perceptual decision-making (Bang and Fleming, 2018). We note, however, that parametric relationships with confidence in this meta-analysis may be due to a particular brain region tracking variables such as response time or stimulus difficulty that themselves covary with confidence, and we are unable to rule out the contribution of these covariates to these results.

A key aspect of metacognition is the extent to which judgements track objective performance, known as metacognitive sensitivity. Sensitivity is defined as the association between performance and confidence over multiple trials and is typically measured using individual-difference metrics such as area under the type 2 receiver operating characteristic curve (AUROC2) or meta-*d’* (Fleming and Lau, 2014). Measures of metacognitive sensitivity are affected by task performance (Galvin et al., 2003; Maniscalco and Lau, 2012), making it important to control for differences in task performance either in the design of experiments (e.g. by using staircase procedures) or in analysis by computing metrics such as metacognitive efficiency (meta-*d’*/*d’*).

In the current meta-analysis, 9 of 11 studies reporting neural correlates of metacognitive sensitivity controlled for performance either in the design of the experiment or in data analysis. Unfortunately, this small sample was likely underpowered for the purposes of the current meta-analysis (Muller et al., 2018), and no significant clusters were observed after correction for multiple comparisons. However, at uncorrected thresholds, we observed involvement of a right aPFC region that was not observed in the parametric confidence meta-analysis (Figure S1). This pattern may indicate that the aPFC plays a role downstream of confidence formation – instead of monitoring performance, aPFC may contribute to updating a mapping between an internal feeling of confidence and the usage of confidence in communication or subsequent control of behaviour (Shekhar and Rahnev, 2018). However, further studies of the neural basis of metacognitive sensitivity (as opposed to confidence level per se) are required to test this hypothesis. The small number of studies reporting sensitivity analyses meant that we were also unable to establish potential domain-specific differences in the neural basis of metacognitive sensitivity, although recent studies have highlighted a specific contribution of precuneus to metamemory sensitivity (Baird et al., 2013, 2015; Mccurdy et al., 2013; Ye et al., 2018).

### Comparing metamemory and metadecision

We observed common regions in separate analyses of metamemory and metadecision tasks, including insula, lateral PFC, and posterior mPFC, suggesting common inputs may drive judgements in both domains (Morales et al., 2018). This metamemory network is similar to that identified by White et al. (2014) in a meta-analysis of nine studies examining retrospective confidence judgements about memory. We also observed partially distinct networks engaged during metacognition of decision-making and memory tasks. Specific to the metamemory analysis were activations in left dorsolateral PFC and clusters in bilateral parahippocampal cortex, whereas specific to metadecision was the involvement of right anterior dorso lateral PFC.

### Temporal focus of metamemory judgements

When separating metamemory judgements by temporal focus, retrospective metamemory activated bilateral parahippocampal cortex and left inferior frontal gyrus, whereas prospective metamemory activated posterior mPFC, left dorsolateral PFC, and right insula. Observing parahippocampal cortex activation for retrospective metamemory and PFC activation for prospective metamemory is consistent with elements of both direct access and inferential accounts of how metacognitive judgements about memory are formed (Metcalfe and Dunlosky, 2008). On one hand, fMRI activation and single-unit responses in the MTL have been linked not only to objective recognition performance (Kao et al., 2005) but also memory confidence (Rutishauser et al., 2015), and feelings of familiarity (Haskins et al., 2008; Henson et al., 2003; Montaldi et al., 2006), consistent with a first-order contribution of mnemonic representations to metacognitive judgement. In contrast, medial prefrontal activation covaries with JOLs independently of first-order performance (Kao et al., 2005) and PFC lesions impair JOL accuracy but not performance (Schnyer et al., 2004), potentially consistent with an inferential basis for prospective confidence.

### Comparing metacognition and mentalising

An appealing model is that metacognition and ToM share a common computational basis that involves recursive inference about our own and others’ mental states. Neural processes supporting ToM are typically assessed by asking subjects to read stories that describe a character’s true or false beliefs while undergoing functional brain imaging (Saxe et al., 2006). These studies have led to the identification of a network encompassing dorsomedial PFC, TPJ, and precuneus as involved in ToM (Amodio and Frith, 2006; Frith and Frith, 1999; Molenberghs et al., 2016; Schurz et al., 2014). However, despite surface similarities in activation location (e.g. precuneus), up until recently, the overlap between large-scale brain networks involved in metacognition and ToM has remained unclear. A notable exception is a study by Valk et al. (2016), who analysed individual differences in cortical thickness and white matter anisotropy related to metacognitive sensitivity on perceptual and higher-order cognitive tasks. It was found that medial prefrontal regions, in which cortical thickness predicted metacognitive ability, overlapped with those from neuroimaging meta-analyses of mentalising.

Here, we assess overlap between our composite meta-analysis of metacognition-related activations and a meta-analysis of mentalising obtained from Neurosynth. While a similar midline network was engaged in both cases, there was in fact minimal overlap between the maps in posterior mPFC and precuneus; instead, metacognition engaged more dorsal and posterior regions. However, overlap was observed in ventromedial and anterior dorsomedial PFC. vmPFC has been specifically associated with self-reflective processing (D’Argembeau et al., 2007; Jenkins and Mitchell, 2011), and its role in ToM tasks is thought to support a simulation of what oneself would do in another’s situation (Jenkins et al., 2008). Intriguingly, in contrast, anterior dorsomedial PFC has been suggested to support second-order representations of mental states, irrespective of whether they originate from self or other (Nicolle et al., 2012; Yoshida et al., 2010). Unique activations for metacognition were observed in insula and lateral PFC, perhaps reflecting the specific contribution of interoception/error monitoring and the formation of confidence estimates, respectively, during self-directed judgements. ToM, in contrast, was uniquely associated with activations in TPJ and temporal pole, consistent with previous findings that these regions are biased towards other-referential processing (Saxe et al., 2006).

These data may tentatively speak to the difference between conceptual and non-conceptual forms of metacognition (Arango-Muñoz, 2011; Proust, 2013). It is plausible that a subset of the unique regions associated with metacognition here (e.g. posterior mPFC and insula) mediate non-conceptual, lower-level epistemic feelings of uncertainty. This perspective is consistent with these regions being found in our parametric confidence meta-analysis activations (Figure 2). In contrast, mPFC may support a conceptual second-order representation of one’s own mental states (conscious elaboration of epistemic feelings) and, in doing so, co-opt similar neural machinery to that engaged when reflecting on or inferring the mental states of others (Carruthers, 2009, 2011; Lombardo et al., 2010). A strong test of this hypothesis would be to compare neural correlates of explicit and implicit measures of self-directed metacognition (Logan and Crump, 2010) with activity engaged when mentalising about others. We would predict that only variation in explicit metacognitive judgements would share commonalities with ToM. However, it is also likely that differences in content between typical ToM and metacognition studies may drive the differences observed here (e.g. judging another person’s emotions or social intentions vs judging one’s own cognitive or decision processes). Further within-subject studies are needed with matched task domain/stimulus materials to draw strong conclusions about the relation between the neural substrates of self- and other-directed metacognition.

### Implications of domain-specific differences in metacognition for neuropsychiatry

One implication of domain-specific neural correlates of metacognition is that damage or disorder affecting these regions may help explain the various types of introspective deficits observed in neuropsychiatry (David et al., 2014). The level of insight into one’s symptoms in schizophrenia have been linked to metacognitive ability over and above differences in executive function (Gilleen et al., 2016), and previous studies of lack of insight have highlighted similar regions to those identified here, including mPFC (van der Meer et al., 2013), insula (Spalletta et al., 2014), inferior frontal gyrus (Orfei et al., 2012), and dorsolateral PFC (Shad, 2004). Individuals with addictions, and those in remission from addiction, have been found to have deficits in metacognition which were predicted by loss of structural integrity in mPFC (Moeller et al., 2016). Furthermore, change in mPFC function has been found to predict the severity and prognosis of addictions (Moeller and Goldstein, 2014).

Neurodegenerative disorders have also been known to bring about progressive anosognosia, or symptom unawareness (García-Cordero et al., 2016). Specifically, Alzheimer’s disease has been associated with metamemory deficits independent of memory deficits, with various studies finding both better and worse insight relative to performance (McGlynn and Kaszniak, 1991; Moulin et al., 2003; Souchay, 2007). The parahippocampal cortex, found in our meta-analysis of retrospective metamemory, is known to be one of the earliest affected regions in the typical progression of Alzheimer’s, potentially consistent with behavioural observations of metamemory deficits (Cosentino, 2014; Didic et al., 2011).

### Analysis limitations

Our study represents a first attempt to consolidate and synthesise findings in the neuroimaging literature on metacognitive judgements and is accompanied by several limitations. First, coordinate-based meta-analyses inevitably sacrifice experimental control to allow aggregating over studies. We were unable to balance the types of tasks (e.g. visual and semantic) used most frequently in different domains, and such imbalances may affect our results. For instance, metadecision studies are typically conducted using visual perceptual tasks, which may bias our results towards this modality. Related to this, most studies in our corpus only examined one particular domain. Because of this, and prominent differences between metamemory and metadecision tasks (i.e. stimulus type), it is not possible to estimate the extent to which differences between domains are related to differences in task. Inferences on parameteric confidence level–related activations are also limited by not incorporating the directionality (e.g. high >low confidence) of the contrast. Second, our analyses collapse across many different judgement types (e.g. FOKs, confidence ratings, and JOLs) that may affect our results if each judgement relies on different processes (Chua et al., 2009; Leonesio and Nelson, 1990; Metcalfe and Dunlosky, 2008). Notably, in metadecision, all judgements were retrospective, so we are unable to assess whether temporality may differentiate metacognitive judgements more generally, or only within metamemory judgements. Finally, all contrasts in our study were univariate, whereas domain-specific differences in confidence-related activation have recently been associated with multivariate patterns of activation in PFC (Morales et al., 2018).

## Conclusion

Despite metacognition occupying a central role in human cognition, the relevant neurocognitive architecture has remained underdetermined, partly due to study-specific differences in both domain and type of metacognitive judgement under study. We used quantitative ALE methods to synthesise findings from 47 neuroimaging studies on metacognition, divided into categories based on the target of metacognitive evaluation (memory and decision-making), analysis type, and, for metamemory judgements, temporal focus (prospective and retrospective). We find engagement of mPFC and lateral PFC, precuneus, and insula in tracking the level of confidence in self-performance of both decision-making and memory tasks, suggesting domain-general contributions to metacognitive judgements. We find, however, preferential engagement of parahippocampal cortex in metamemory experiments and right anterior dorsolateral PFC in metadecision experiments. Finally, by comparing our results to comparable analyses of mentalising, we obtain evidence for common engagement of the ventromedial and anterior dorsomedial PFC in metacognition and mentalising, suggesting that these regions may support second-order representations for thinking about the thoughts of oneself and others.

## Supplemental Material

VaccaroFleming_SupplementaryMaterials – Supplemental material for Thinking about thinking: A coordinate-based meta-analysis of neuroimaging studies of metacognitive judgements Click here for additional data file. Supplemental material, VaccaroFleming_SupplementaryMaterials for Thinking about thinking: A coordinate-based meta-analysis of neuroimaging studies of metacognitive judgements by Anthony G. Vaccaro and Stephen M. Fleming in Brain and Neuroscience Advances
