# Supplementary material for: Thinking about thinking: A coordinate-based meta-analysis of neuroimaging studies of metacognitive judgements
Source: Brain Neurosci Adv. 2018 Nov 13;2:2398212818810591. doi: 10.1177/2398212818810591 (PMC6238228; doi:10.1177/2398212818810591)
Supplement: VaccaroFleming_SupplementaryMaterials – Supplemental material for Thinking about thinking: A coordinate-based meta-analysis of neuroimaging studies of metacognitive judgements [file VaccaroFleming_SupplementaryMaterials.pdf]

Supplementary Materials

Table S1. Table of publications included in the meta-analysis

Reference	N subjects	Contrast	Domain	Temporal focus	Analysis type	Judgment type	Sign of parametric activation / correlation	Structural/ functional	MNI/T88
Allen, M., Glen, J. C., Müllensiefen, D., Schwarzkopf, D. S., Callaghan, M. F., & Rees, G. (2017). Metacognitive ability predicts hippocampal and prefrontal microstructure. *NeuroImage*, 149*(1)*, 415–423.	48	Whole Brain Results, Table 2	Decision	Retrospective	Metacognitive sensitivity	Confidence	N/A	Struc	MNI
Baird, B., Smallwood, J., Gorgolewski, K. J., & Margulies, D. S. (2013). Medial and Lateral Networks in Anterior Prefrontal Cortex Support Metacognitive Ability for Memory and Perception. *Journal of Neuroscience*, 33*(42)*, 16657–16665.	52	Connectivity with medial and lateral aPFC for memory, Table 1	Memory	Retrospective	Metacognitive sensitivity	Confidence	Pos	Func	MNI
		Connectivity with medial and lateral aPFC for perception, Table 1	Decision	Retrospective	Metacognitive sensitivity	Confidence	Pos	Func	MNI
		Differential connectivity with medial and lateral aPFC, Table 2	Memory	Retrospective	Metacognitive sensitivity	Confidence	N/A	Func	MNI
Baird, B., Cieslak, M., Smallwood, J., Grafton, S. T., & Schooler, J. W. (2015). Regional white matter variation associated with domain- specific metacognitive accuracy. *Journal of Cognitive Neuroscience*, 27*(3)*, 440–452.	38	White matter microstructure associated with metacognitive ability in perception, Table 1	Decision	Retrospective	Metacognitive sensitivity	Confidence	Pos	Struc	MNI
		White matter microstructure associated with metacognitive ability in memory, Table 1	Memory	Retrospective	Metacognitive sensitivity	Confidence	Pos	Struc	MNI
Chen, J., Feng, T., Shi, J., Liu, L., & Li, H. (2013). Neural representation of decision confidence. *Behavioural Brain Research*, 245, 50–57.	20	Brain regions exhibiting significant activation in association with decision confidence process, Table 1	Decision	Retrospective	Parametric	Confidence	Pos, Neg	Func	T88
Chua, C., Rand-Giovannetti, E., Schacter, D. L., Albert, M. S., Chua, E. F., & Sperling, R. A. (2004). Dissociating Confidence and Accuracy: Functional Magnetic Resonance Imaging Shows Origins of the Subjective Memory Experience. *Journal of Cognitive Neuroscience*, 16*(7)*, 1131–1142.	16	HC correct vs. LC correct, Table 3	Memory	Retrospective	Parametric	Confidence	Pos	Func	MNI
		HC incorrect vs. LC incorrect, Table 3	Memory	Retrospective	Parametric	Confidence	Pos	Func	MNI

Chua, E. F., Schacter, D. L., Rand-Giovannetti, E., & Sperling, R. A. (2006). Understanding metamemory: Neural correlates of the cognitive process and subjective level of confidence in recognition memory. *NeuroImage*, 29.	20	Conf HC Correct> Conf LC Correct, Table 2	Memory	Retrospective	Parametric	Confidence	Pos	Func	MNI
		Conf HC Incorrect> Conf LC Incorrect, Table 2	Memory	Retrospective	Parametric	Confidence	Pos	Func	MNI
		Confidence assessment>recognition, Table 2	Memory	Retrospective	Judgement-Related	Confidence	N/A	Func	MNI
Chua, E. F., Schacter, D. L., & Sperling, R. A. (2009). Neural basis for recognition confidence in younger and older adults. *Psychology and Aging*, 24*(1)*, 139–153.	16	Regions significant for HC in young but not old, Table 3	Memory	Retrospective	Parametric	Confidence	Pos	Func	MNI
		Regions significant for LC compared to HC, Table 2	Memory	Retrospective	Parametric	Confidence	Neg	Func	MNI
Chua, E. F., Schacter, D. L., & Sperling, R. A. (2009). Neural correlates of metamemory: a comparison of feeling-of-knowing and retrospective confidence judgments. *Journal of Cognitive Neuroscience*, 21*(9)*, 1751–65.	13	Metamemory>Non-metamemory, Table 2	Memory	Both grouped together	Judgement-Related	Feeling of Knowing+Confidence	N/A	Func	MNI
		Metamemory>Recognition, Table 2	Memory	Both grouped together	Judgement-Related	Feeling of Knowing+Confidence	N/A	Func	MNI
		FOK> non-metamemory, Table 2	Memory	Prospective	Judgement-Related	Feeling of Knowing	N/A	Func	MNI
De Martino, B., Fleming, S. M., Garrett, N., & Dolan, R. J. (2013). Confidence in value-based choice. *Nature Neuroscience*, 16*(1)*, 105–10.	20	Confidence increase, Supplementary Table 3	Decision	Retrospective	Parametric	Confidence	Pos	Func	MNI
		Confidence decrease, Supplementary Table 3	Decision	Retrospective	Parametric	Confidence	Pos	Func	MNI
Do Lam, A. T. A., Axmacher, N., Fell, J., Staresina, B. P., Gauggel, S., Wagner, T., Olligs, J. & Weis, S. (2012). Monitoring the Mind: The Neurocognitive Correlates of Metamemory. *PLoS ONE*, 7*(1)*, e30009.	17	JOL predicting memory formation, Table 1	Memory	Prospective	Parametric	Judgement of Learning	N/A	Func	MNI
Elman, J. A., Klostermann, E. C., Marian, D. E., Verstaen, A., & Shimamura, A. P. (2012). Neural correlates of metacognitive monitoring during episodic and semantic retrieval. *Cognitive, Affective, & Behavioral Neuroscience*, 12*(3)*, 599–609.	19	Episodic Definitely>Likely/Maybe, Table 3	Memory	Prospective	Parametric	Feeling of Knowing	Pos	Func	MNI
		Semantic Definitely>Likely/Maybe, Table 3	Memory	Prospective	Parametric	Feeling of Knowing	Pos	Func	MNI
		Episodic Likely/Maybe>Definitely, Table 4	Memory	Prospective	Parametric	Feeling of Knowing	Pos	Func	MNI
		Semantic Likely/Maybe>Definitely, Table 4	Memory	Prospective	Parametric	Feeling of Knowing	Pos	Func	MNI

Fleming, S. M., Weil, R. S., Nagy, Z., Dolan, R. J., & Rees, G. (2010). Relating introspective accuracy to individual differences in brain structure. *Science*, 329*(5998)*, 1541–1543.	31	FA associated with A_{roc}, Table S3	Decision	Retrospective	Metacognitive sensitivity	Confidence	Pos/Neg	Struct	MNI
		GM associated with A_{roc}, Table S2	Decision	Retrospective	Metacognitive sensitivity	Confidence	Pos/Neg	Struct	MNI
Fleming, S. M., Huijgen, J., & Dolan, R. J. (2012). Prefrontal Contributions to Metacognition in Perceptual Decision Making. *Journal of Neuroscience*, 32*(18)*, 6117–6125.	23	Report>Follow, Table 1	Decision	Retrospective	Judgement-Related	Confidence	N/A	Func	MNI
		Correlation with reported confidence, Table 2	Decision	Retrospective	Parametric	Confidence	Neg	Func	MNI
Harsay, H. A., Spaan, M., Wijnen, J. G., & Ridderinkhof, K. R. (2012). Error Awareness and Salience Processing in the Oddball Task: Shared Neural Mechanisms. *Frontiers in Human Neuroscience*, 6, 246.	14	Complete list of areas with significant BOLD during aware versus unaware errors, Table A1	Decision	Retrospective	Parametric	Aware Error	N/A	Func	MNI
Hayes, S. M., Buchler, N., Stokes, J., Kragel, J., & Cabeza, R. (2011). Neural correlates of confidence during item recognition and source memory retrieval: evidence for both dual-process and strength memory theories. *Journal of Cognitive Neuroscience*, *23*(12), 3959-3971.	16	High> Low confidence, Table 2	Memory	Retrospective	Parametric	Confidence	Pos	Func	MNI
		Low>High confidence, Table 2	Memory	Retrospective	Parametric	Confidence	Pos	Func	MNI
Hebart, M. N., Schriever, Y., Donner, T. H., & Haynes, J.-D. (2016). The Relationship between Perceptual Decision Variables and Confidence in the Human Brain. *Cerebral Cortex* , 26*(1)*, 118–130.	18	Brain regions where activity is related to confidence, Table 1	Decision	Retrospective	Parametric	Confidence	Pos, Neg	Func	MNI
Heereman, J., Walter, H., & Heekeren, H. R. (2015). A task-independent neural representation of subjective certainty in visual perception. *Frontiers in Human Neuroscience*, 9, 551.	20	Significant correlations with subjective certainty in color trials, Table 2	Decision	Retrospective	Parametric	Confidence	Pos, Neg	Func	MNI
		Significant correlations with subjective certainty in motion trials, Table 3	Decision	Retrospective	Parametric	Confidence	Pos, Neg	Func	MNI
		Task-independent significant correlations with subjective certainty, Table 4	Decision	Retrospective	Parametric	Confidence	Pos, Neg	Func	MNI
Henson, R. N., Rugg, M. D., Shallice, T., & Dolan, R. J. (2000). Confidence in Recognition Memory for Words: Dissociating Right Prefrontal Roles in Episodic Retrieval. *Journal of*	12	Low > High Confidence, Figure 2	Memory	Retrospective	Parametric	Confidence	Pos	Func	MNI

Cognitive Neuroscience, 12*(6)*, 913–923.									
Hester, R., Foxe, J. J., Molholm, S., Shpaner, M., & Garavan, H. (2005). Neural mechanisms involved in error processing: A comparison of errors made with and without awareness. *NeuroImage*, 27, 602–608	13	Event-related activation during aware errors, Table 1	Decision	Retrospective	Parametric, Judgement-Related	Aware Error	N/A	Func	MNI
Hester, R., Nestor, L., & Garavan, H. (2009). Impaired Error Awareness and Anterior Cingulate Cortex Hypoactivity in Chronic Cannabis Users. *Neuropsychopharmacology*, 34*(11)*, 2450–2458.	32	Aware errors>unaware errors, Table 3	Decision	Retrospective	Parametric, Judgement-Related	Aware Error	N/A	Func	MNI
Hilgenstock, R., Weiss, T., & Witte, O. W. (2014). You'd Better Think Twice: Post-Decision Perceptual Confidence. *NeuroImage*, 99, 323–331	24	Regions involved in decisional confidence processing, Table 1	Decision	Retrospective	Parametric	Confidence	Neg	Func	MNI
Huijbers, W., Papp, K. V., LaPoint, M., Wigman, S. E., Dagley, A., Hedden, T., Rentz, D.M., Schultz, A.P. & Sperling, R. A. (2016). Age-Related Increases in Tip-of-the-tongue are Distinct from Decreases in Remembering Names: A Functional MRI Study. *Cerebral Cortex*, 1*(11)*.	73	TOT > (REC +FOK + UNK), Figure 3C	Memory	Prospective	Parametric	Tip of the Tongue	Pos	Func	MNI
Kao, Y.-C., Davis, E. S., & Gabrieli, J. D. E. (2005). Neural correlates of actual and predicted memory formation. *Nature Neuroscience*, 8*(12)*, 1776–1783.	16	Predicted encoding success, Table 1	Memory	Prospective	Parametric	Judgement of Learning	Pos	Func	MNI
Kikyo, H., Ohki, K., & Sekihara, K. (2001). Temporal characterization of memory retrieval processes: an fMRI study of the 'tip of the tongue'phenomenon. *European Journal of Neuroscience*, *14*(5), 887-892.	14	Hit on>give up, Table 1	Memory	Prospective	Parametric	Tip of the Tongue	Pos	Func	MNI
Kikyo, H., Ohki, K., & Miyashita, Y. (2002). Neural Correlates for Feeling-of-Knowing: An fMRI Parametric Analysis. *Neuron*, 36*(1)*, 177–186	14	Regions parametrically modulated by FOK with double parametric analysis, Table 2	Memory	Prospective	Parametric	Feeling of Knowing	Pos	Func	MNI
Kikyo, H., & Miyashita, Y. (2004). Temporal lobe activations of "feeling-of-knowing" induced by face-name associations. *NeuroImage*, 23*(4)*, 1348–1357.	15	Brain regions parametrically modulated by the FOK, Table 1	Memory	Prospective	Parametric	Feeling of Knowing	Pos	Func	MNI
Kim, H., & Cabeza, R. (2007). Trusting Our Memories: Dissociating the Neural Correlates of Confidence in	11	HC-TR> LC-TR and LC-FR >HC-FR, Table 3	Memory	Retrospective	Metacognitive sensitivity	Confidence	Pos, Neg	Func	T88
		LC-TR> HC-TR and HC-	Memory	Retrospective	Metacognitive	Confidence	Pos, Neg	Func	T88

Veridical versus Illusory Memories. *Journal of Neuroscience*, 27*(45)*.		FR> LC-FR, Table 3			Sensitivity				
		True Recognition High-confidence> Low-Confidence, Table 2	Memory	Retrospective	Parametric	Confidence	Pos	Func	T88
		True Recognition Low-confidence> High confidence, Table 2	Memory	Retrospective	Parametric	Confidence	Pos	Func	T88
		False Recognition High confidence> Low confidence, Table 2	Memory	Retrospective	Parametric	Confidence	Pos	Func	T88
		False Recognition Low confidence> High confidence, Table 2	Memory	Retrospective	Parametric	Confidence	Pos	Func	T88
Kim, H., & Cabeza, R. (2009). Common and specific brain regions in high- versus low-confidence recognition memory. *Brain Research*, 1282, 103–13.	12	HCR activity, Table 2	Memory	Retrospective	Parametric	Confidence	Pos	Func	T88
		LCR activity, Table 2	Memory	Retrospective	Parametric	Confidence	Pos	Func	T88
Klein, T. A., Endrass, T., Kathmann, N., Neumann, J., von Cramon, D. Y., & Ullsperger, M. (2007). Neural correlates of error awareness. *Neuroimage*, *34*(4), 1774-1781.	13	Conscious error awareness, Table 3	Decision	Retrospective	Parametric	Aware Error	Pos	Func	T88
Kuchinke, L., Fritzemeier, S., Hofmann, M. J., & Jacobs, A. M. (2013). Neural correlates of episodic memory: associative memory and confidence drive hippocampus activations. *Behavioural brain research*, *254*, 92-101.	20	Significantly greater BOLD for high and low confidence, Table 4	Memory	Retrospective	Parametric	Confidence	Pos	Func	MNI
Maril, A., Wagner, A. D., & Schacter, D. L. (2001). On the tip of the tongue: An event-related fMRI study of semantic retrieval failure and cognitive conflict. *Neuron*, *31*(4), 653-660.	14	TOT-Selective Response, Figure 1	Memory	Prospective	Parametric	Tip of the Tongue	Pos	Func	MNI
Maril, A., Simons, J. S., Mitchell, J. P., Schwartz, B. L., & Schacter, D. L. (2003). Feeling-of-knowing in episodic memory: an event-related fMRI study. *Neuroimage*, *18*(4), 827-836.	17	K>FOK>DK, Table 2	Memory	Prospective	Parametric	Feeling of Knowing	Pos	Func	MNI
		K=FOK>DK, Table 2	Memory	Prospective	Parametric	Feeling of Knowing	Pos	Func	MNI
Maril, A., Simons, J. S., Weaver, J. J., & Schacter, D. L. (2005). Graded recall success: An event-related fMRI	15	TOT>all else, Table 3	Memory	Prospective	Parametric	Tip of the Tongue	N/A	Func	MNI

comparison of tip of the tongue and feeling of knowing. *Neuroimage*, *24*(4), 1130-1138.		TOT=FOK>all else, Table 3	Memory	Prospective	Parametric	Tip of the Tongue	N/A	Func	MNI
McCurdy, L. Y., Maniscalco, B., Metcalfe, J., Liu, K. Y., de Lange, F. P., & Lau, H. (2013). Anatomical coupling between distinct metacognitive systems for memory and visual perception. *Journal of Neuroscience*, *33*(5), 1897-1906.	34	meta-d'/d' for vision	Decision	Retrospective	Metacognitive sensitivity	Confidence	Pos	Struc	MNI
		meta-d'/d' for memory	Memory	Retrospective	Metacognitive sensitivity	Confidence	Pos	Struc	MNI
Moritz, S., Gläscher, J., Sommer, T., Büchel, C., & Braus, D. F. (2006). Neural correlates of memory confidence. *Neuroimage*, *33*(4), 1188-1193.	17	Activation pattern for high-confidence vs. low-confidence responses, Table 1	Memory	Retrospective	Parametric	Confidence	Pos	Func	MNI
		Low-confidence vs. high-confidence, Table 1	Memory	Retrospective	Parametric	Confidence	Pos	Func	MNI
Orr, C., & Hester, R. (2012). Error-related anterior cingulate cortex activity and the prediction of conscious error awareness. *Frontiers in human neuroscience*, *6*, 177.	56	Greater BOLD for aware than unaware errors, Table 2	Decision	Retrospective	Parametric, Judgement-Related	Aware Error	Pos	Func	MNI
Paul, E. J., Smith, J. D., Valentin, V. V., Turner, B. O., Barbey, A. K., & Ashby, F. G. (2015). Neural networks underlying the metacognitive uncertainty response. *Cortex*, *71*, 306-322.	29	Uncertainty>correct categorization contrast, Table 3	Decision	Retrospective	Judgement-Related	Uncertainty Response	N/A	Func	MNI
Plailly, J., Tillmann, B., & Royet, J. P. (2007). The feeling of familiarity of music and odors: the same neural signature?. *Cerebral cortex*, *17*(11), 2650-2658.	13	Significant for bimodal familiar compared to unfamiliar, Table 1	Memory	Retrospective	Parametric	Feeling of Familiarity	Pos	Func	MNI
		Bimodal activations evoked by feelings of unfamiliarity, Table 2	Memory	Retrospective	Parametric	Feeling of Familiarity	Neg	Func	MNI
Reggev, N., Zuckerman, M., & Maril, A. (2011). Are all judgments created equal?: An fMRI study of semantic and episodic metamemory predictions. *Neuropsychologia*, *49*(5), 1332-1342.	18	Gray matter regions modulated by predictive judgements, Table 3	Memory	Prospective	Parametric	Feeling of Knowing	Pos	Func	MNI
Risius, U. M., Staniloiu, A., Piefke, M., Maderwald, S., Schulte, F., Brand, M., & Markowitsch, H. J. (2013). Retrieval, monitoring, and control processes: a 7 tesla FMRI approach to memory accuracy. *Frontiers in*	29	High confidence>Low confidence, Table 2 (A)	Memory	Retrospective	Parametric	Confidence	Pos	Func	MNI

behavioral neuroscience, *7*, 24.		Low confidence>High confidence, Table 2 (B)	Memory	Retrospective	Parametric	Confidence	Pos	Func	MNI
		Monitoring greater than retrieval, Table 4	Memory	Retrospective	Judgement-Related	Confidence	N/A	Func	MNI
Schnyer, D. M., Nicholls, L., & Verfaellie, M. (2005). The role of VMPC in metamemorial judgments of content retrievability. *Journal of cognitive neuroscience*, *17*(5), 832-846.	19	Brain regions linearly modulated by 1-4 rating, Table 3	Memory	Prospective	Parametric	Feeling of Knowing	Pos	Func	MNI
		Regions associated with all accurate retrieval judgements, Table 2 (c)	Memory	Prospective	Metacognitive sensitivity	Feeling of Knowing	N/A	Func	MNI
Sinanaj, I., Cojan, Y., & Vuilleumier, P. (2015). Inter-individual variability in metacognitive ability for visuomotor performance and underlying brain structures. *Consciousness and Cognition*, *36*, 327-337.	29	Gray matter positive correlation with Mratio, Figure 3	Decision	Retrospective	Metacognitive sensitivity	Confidence	Pos	Struc	MNI
		Gray matter negative correlation with MRatio	Decision	Retrospective	Metacognitive sensitivity	Confidence	Neg	Struc	MNI
Valk, S. L., Bernhardt, B. C., Böckler, A., Kanske, P., & Singer, T. (2016). Substrates of metacognition on perception and metacognition on higher-order cognition relate to different subsystems of the mentalizing network. *Human Brain Mapping*, *37*(10), 3388-3399.	151	MC-P: Cortical thickness, Supplementary Table 1	Decision	Retrospective	Metacognitive sensitivity	Confidence	N/A	Struc	MNI

		MC-P Fractional anisotropy, Supplementary Table 1	Decision	Retrospective	Metacognitive sensitivity	Confidence	N/A	Struc	MNI
Wan, X., Cheng, K., & Tanaka, K. (2016). The Neural System of Postdecision Evaluation in Rostral Frontal Cortex during Problem-solving Tasks. *eNeuro*, *3*(4), ENEURO-0188.	34	Post-task activation, Table 1	Decision	Retrospective	Judgement-Related	Confidence	N/A	Func	T88
Wu, S. W., Delgado, M. R., & Maloney, L. T. (2015). Gambling on visual performance: neural correlates of metacognitive choice between visual lotteries. *Frontiers in Neuroscience*, *9*, 314.	19	Reward probability, Table 1	Decision	Retrospective	Parametric	Lottery	Pos	Func	MNI
Yang, H., Cai, Y., Liu, Q., Zhao, X., Wang, Q., Chen, C., & Xue, G. (2015). Differential neural correlates underlie judgment of learning and subsequent memory performance. *Frontiers in Psychology*, *6*, 1699.	26	Higher JOL, Figure 3	Memory	Prospective	Parametric	Judgement of Learning	Pos	Func	MNI
		Effect of JOL accuracy on subsequent JOL effect, Figure 4	Memory	Prospective	Metacognitive Sensitivity	Gamma	Neg	Func	MNI
		PPI analysis higher JOL, Figure 6C	Memory	Prospective	Parametric	Judgement of Learning	Pos, Neg	Func	MNI
		Difference between memory and JOL effects, Figure 6D	Memory	Prospective	Judgement-Related	Judgement of Learning	N/A	Func	MNI

Yokoyama, O., Miura, N., Watanabe, J., Takemoto, A., Uchida, S., Sugiura, M., Horie, K., Sato, S., Kawashima, R. & Nakamura, K. (2010). Right frontopolar cortex activity correlates with reliability of retrospective rating of confidence in short-term recognition memory performance. *Neuroscience Research*, *68*(3), 199-206.	25	Higher activity for confidence rating than brightness discrimination, Table 1	Memory	Retrospective	Judgment-Related	Confidence	N/A	Func	MNI
		Signal change associated with higher gamma, Figure 4	Memory	Retrospective	Metacognitive sensitivity	Confidence	N/A	Func	MNI

Table S2. Uncorrected findings for judgment-related and sensitivity-related contrasts. We applied an uncorrected threshold of p<.001 with a minimum cluster size of 200mm^3.

Cluster	Peak Coordinate (MNI)			Volume (mm^3)	Region	Maximum ALE Value
Judgement	x	y	z			
1	-50	-48	40	592	L Lateral parietal cortex	0.0153
2	0	20	38	576	L/R Posterior medial frontal cortex	0.0161
3	48	-48	40	464	R Lateral parietal cortex	0.0171
4	42	14	2	320	R Insula	0.0135
5	38	42	28	232	R Anterior dorsolateral prefrontal cortex	0.0113
6	26	62	-4	224	R Anterior prefrontal cortex	0.0114
7	-62	-28	20	224	L Superior temporal gyrus	0.0124
Sensitivity						
1	32	50	8	504	R Anterior prefrontal cortex	0.0176
2	-26	-36	-14	448	L Parahippocampal cortex	0.0169
3	30	16	-18	288	R Insula	0.0120
4	8	42	44	272	R Dorsomedial prefrontal cortex	0.0114
**5**	8	-58	20	240	R Precuneus	0.0122

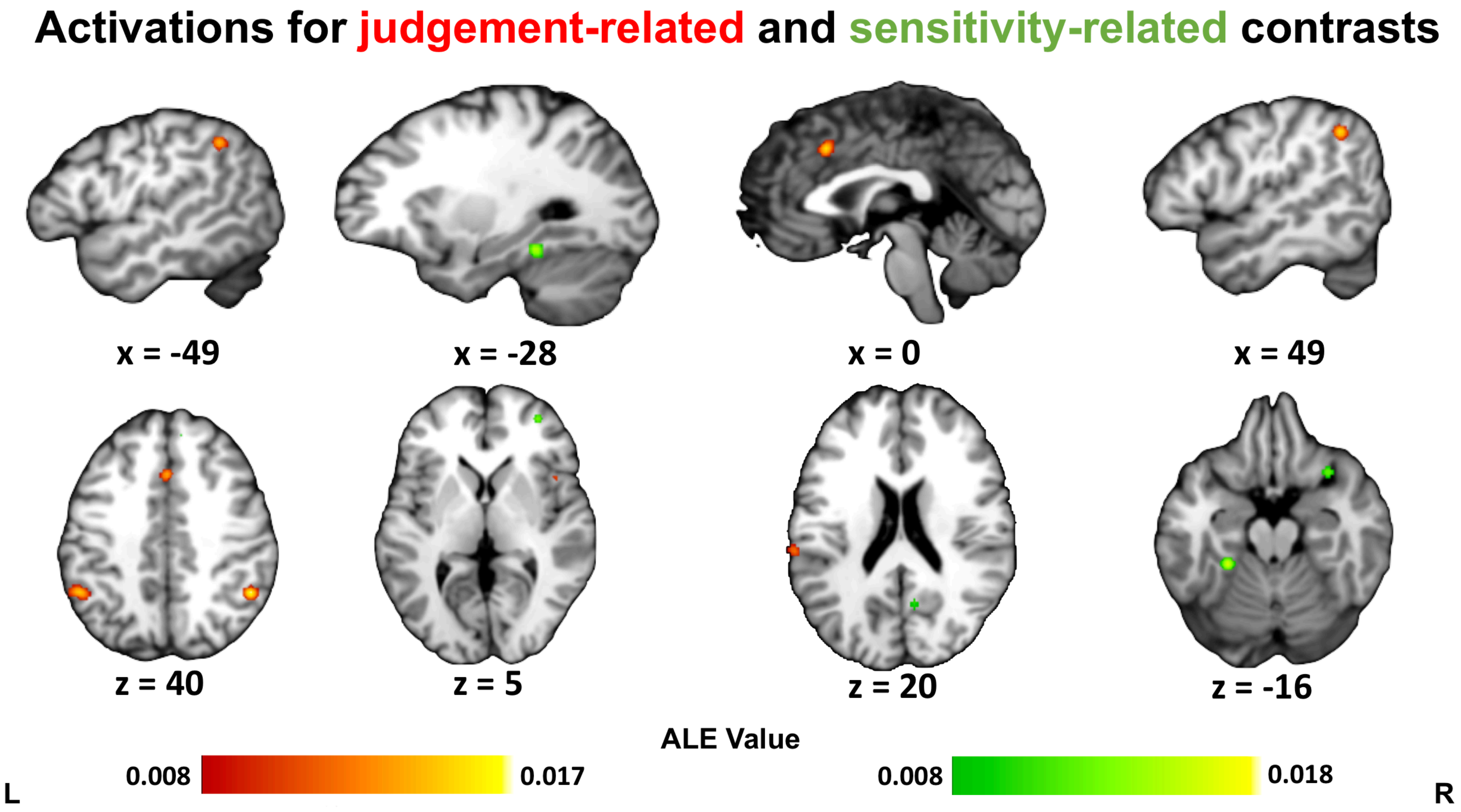

Figure S1. ALE results for contrasts of judgement-related activity (red) and metacognitive sensitivity-related activity (green). Clusters are displayed in MNI standard space. An uncorrected threshold of $p<.001$ was used, with a minimum cluster size of 200mm^3.
